# Perturbation of microRNAs in Rat Heart during Chronic Doxorubicin Treatment

**DOI:** 10.1371/journal.pone.0040395

**Published:** 2012-07-31

**Authors:** Caterina Vacchi-Suzzi, Yasmina Bauer, Brian R. Berridge, Sandrine Bongiovanni, Kevin Gerrish, Hisham K. Hamadeh, Martin Letzkus, Jonathan Lyon, Jonathan Moggs, Richard S. Paules, François Pognan, Frank Staedtler, Martin P. Vidgeon-Hart, Olivier Grenet, Philippe Couttet

**Affiliations:** 1 Discovery and Investigative Safety, Novartis Institutes for Biomedical Research, Basel, Switzerland; 2 Translational Science Biology, Actelion Pharmaceuticals Ltd, Allschwil, Switzerland; 3 Safety Assessment, GlaxoSmithKline, Research Triangle Park, North Carolina, United States of America; 4 National Institute of Environmental Health Science, National Institutes of Health, Research Triangle Park, North Carolina, United States of America; 5 Comparative Biology and Safety Sciences, Amgen Inc., Thousand Oaks, California, United States of America; 6 Biomarker Development, Novartis Institutes for Biomedical Research, Basel, Switzerland; 7 Investigative Preclinical Toxicology, GlaxoSmithKline, Ware, United Kingdom; IRCCS-Policlinico San Donato, Italy

## Abstract

Anti-cancer therapy based on anthracyclines (DNA intercalating Topoisomerase II inhibitors) is limited by adverse effects of these compounds on the cardiovascular system, ultimately causing heart failure. Despite extensive investigations into the effects of doxorubicin on the cardiovascular system, the molecular mechanisms of toxicity remain largely unknown. MicroRNAs are endogenously transcribed non-coding 22 nucleotide long RNAs that regulate gene expression by decreasing mRNA stability and translation and play key roles in cardiac physiology and pathologies. Increasing doses of doxorubicin, but not etoposide (a Topoisomerase II inhibitor devoid of cardiovascular toxicity), specifically induced the up-regulation of miR-208b, miR-216b, miR-215, miR-34c and miR-367 in rat hearts. Furthermore, the lowest dosing regime (1 mg/kg/week for 2 weeks) led to a detectable increase of miR-216b in the absence of histopathological findings or alteration of classical cardiac stress biomarkers. *In silico* microRNA target predictions suggested that a number of doxorubicin-responsive microRNAs may regulate mRNAs involved in cardiac tissue remodeling. In particular miR-34c was able to mediate the DOX-induced changes of Sipa1 mRNA (a mitogen-induced Rap/Ran GTPase activating protein) at the post-transcriptional level and in a seed sequence dependent manner. Our results show that integrated heart tissue microRNA and mRNA profiling can provide valuable early genomic biomarkers of drug-induced cardiac injury as well as novel mechanistic insight into the underlying molecular pathways.

## Introduction

Doxorubicin (DOX), a potent drug used in cancer chemotherapy, intercalates with DNA and stabilizes a ternary complex with Topoisomerase II (Top2) thus preventing replication of DNA and subsequent cell proliferation [1].

Despite its beneficial therapeutic effects, acute administration of high DOX doses causes severe kidney damage, whilst toxic cardiomyopathy is observed when DOX is chronically administered for several weeks at doses devoid of severe nephrotoxicity [2], [3], [4], [5]. The exact mechanism of its cardiac toxicity remains largely unknown. In a clinical setting, the occurrence of DOX-induced cardiomyopathy is mitigated by prophylactic treatment with dexrazoxane (DZR), a Top2 inhibitor and iron-chelating agent which limits the amount of metal ions interacting with DOX and thus reduces oxidative stress [2]. The cardioprotective mechanism of DZR is at present not fully understood [6].

Administration of DOX ranging from 1 to 3 mg/kg/week to rats during several weeks recapitulates the cardiac symptoms observed in humans [7], [8]. Similarly, combined treatment with DZR lessens the cardiac symptoms in rats treated with DOX [9].

MicroRNAs are a class of genomically encoded non-translated short RNAs, about 22 nucleotides long, discovered in plants in 1993 [10]. They are evolutionarily conserved throughout higher eukaryotes, from *C.elegans* to human, and their function is exerted via the mammalian RNA interference machinery [11]. They play a role in the post-transcriptional regulation of target genes, via imperfect base complementarities, and control gene expression mainly via translation inhibition and mRNA degradation [12], [13], [14]. MicroRNAs are involved in a diverse range of physiological and pathological processes including organism development [15], cancer [16] and immunity [17], [18], [19]. In particular, microRNAs are key players in the development, physiology and pathology of the heart [20], [21], [22]. MicroRNAs have been shown to be differentially expressed in response to various signals resulting in post-transcriptional regulation of their predicted target genes. This extra level of regulation is critical during heart development and important in the cardiac response to stress [23], [24].

Recent studies investigating DOX-induced changes in gene expression at the mRNA level in rat cardiac tissue have successfully identified genomic biomarkers whose molecular functions are consistent with proposed toxicity mechanisms [9]. However the emerging importance of microRNAs for the regulation of cardiac physiology and pathologies [20], [21], [22], together with their potential to provide novel insight into mechanisms of xenobiotics-induced toxicity [25], suggest that the integration of mRNA and microRNA profiling may provide new opportunities for elucidating mechanisms of DOX-induced cardiotoxicity.

In the present study, we report that the chronic myocardial toxicity induced by DOX in rats was associated with the modulation of microRNAs, and some of these can be phenotypically anchored to histopathology findings. Alteration of miR-216b could be detected earlier than overt myocardial damage. Furthermore, by integrating DOX-inducible microRNAs with mRNA profiles generated from the same cardiac tissue samples, the putative target genes of the DOX-induced microRNA signature were highlighted, including a number of genes that have not previously been described in DOX-induced cardiomyopathy. Finally, evidence for the direct targeting of Sipa1 mRNAs by miR-34c was provided for the first time, and this microRNA-mRNA interaction could potentially play a role in the molecular response to DOX in the rodent heart.

## Results

### Doxorubicin Induced Cytoplasm Vacuolation and dysregulation of Tissue Genomic Cardiomyopathy Indicators

**Figure 1:**
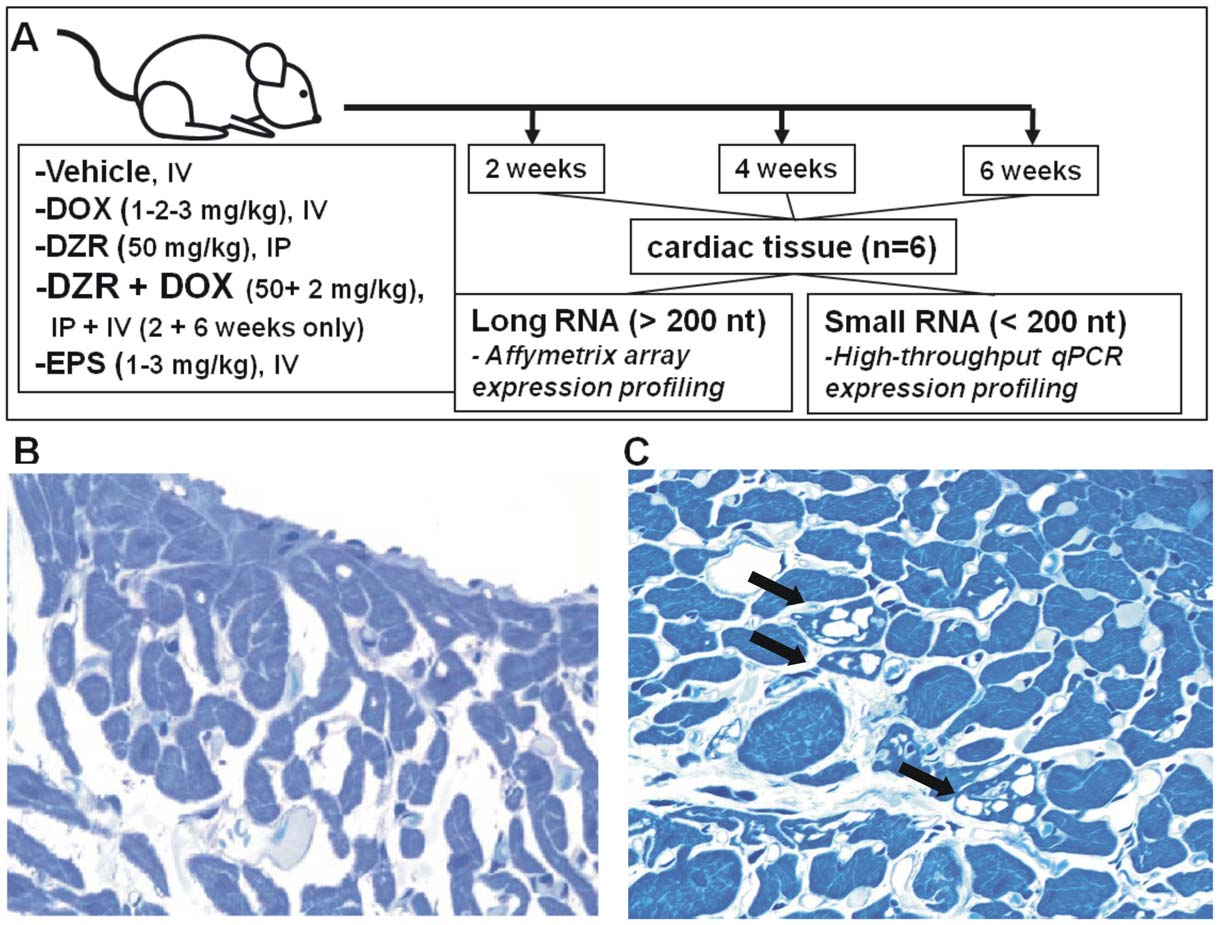
Study design and representative micrograph showing DOX-related vacuolation in the myocardium. (A) Six adult male rats were injected with the indicated doses of vehicle, doxorubicin (DOX), dexrazoxane (DZR), etoposide (EPS) or a combination of DOX and DZR for 2, 4 or 6 weeks. Cardiac tissue was excised and deep frozen for gene expression and microRNA profiling experiments. A representative micrograph of a toluidine blue stained myocardial section of a control (B) and of a DOX treated animal (C). Black arrows indicate sarcoplasmic micro- and macro- vacuolation of cardiomyocytes.

Male rats were treated with DOX according to the scheme reported in Figure 1A. Macro-and micro-vesicular vacuolation of individual cardiac myocytes was observed in atria and ventricles of DOX-treated animals. Representative histopathological image is shown in Figure 1B and C. Atrial and ventricular vacuolation occurred with increasing frequency and severity over time, with or without continued weekly exposure to drug (Results S1 and Table S2). The vacuoles could be caused by autophagic vesicles accumulation [26]; however, a hierarchical clustering using the expression of 31 autophagy-related genes could not discriminate between vehicle and DOX treated cardiac samples (data not shown). Given the small proportion of cardiomyocytes undergoing vacuolation within the whole tissue it is possible that the expression changes of a large list of genes would be diluted by background signals. The treatment of DOXO at 1, 2 or 3 mg/kg/week for 2, 4 and 6 weeks showed a statistically significant increase of Ambra1 mRNA, a positive autophagy regulator [27] but did not affect the level of other markers (see below).

**Figure 2:**
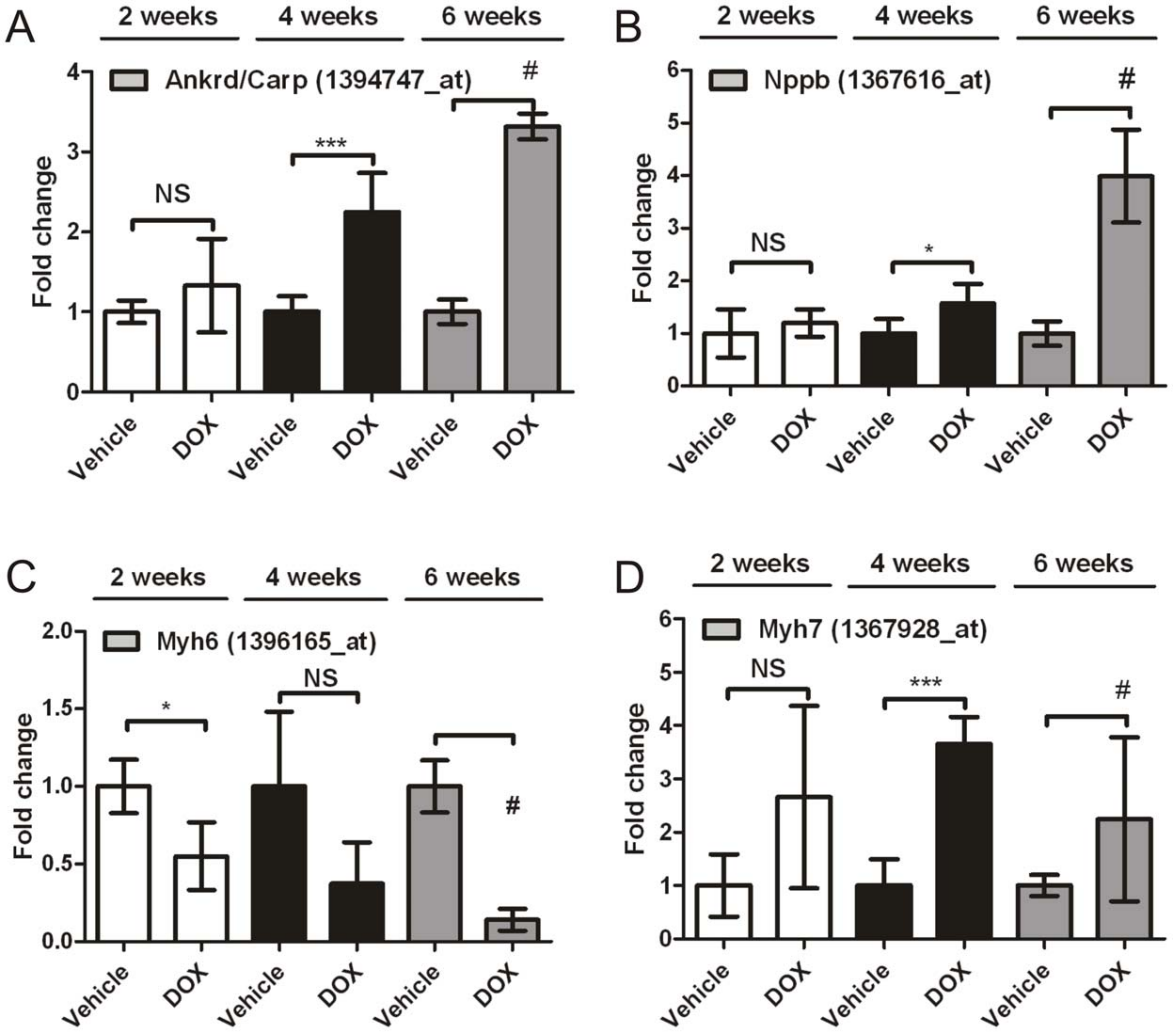
DOX 3 mg/kg/week altered levels of genomic cardiomyopathy indicators (Ankrd/Carp, Nppb, Myh7 and Myh6). Expression fold change relative to vehicle were represented for DOX 3 mg/kg/week at 2, 4 and 6 weeks time point (n = 6) for (A) Ankrd/Carp, (B) Nppb, (C) Myh7 and (D) Myh6. For each time point and each probe set, vehicle values were averaged and normalized to 1. The same correction was applied to the DOX treated values. Affymetrix probe-set number is indicated in brackets. Error bars represent standard deviation. T-test was performed for vehicle- vs. DOX-treated at each time point. *P<0.05, **P<0.01, ***P<0.005, NS = Non-Significant. (No t-test for #, as n = 2).

The administration of DOX at 3 mg/kg/week induced time-dependent changes in the expression of mRNAs that have previously been associated with molecular responses underlying cardiac pathologies [28], [29], [30] (Figure 2). A significant increase of Ankyrin repeat domain 1 (Ankrd1/Carp), natriuretic peptide precursor type B (Nppb) and myosin heavy chain 7 (Myh7/β) mRNA and a significant decrease of myosin heavy chain 6 (Myh6/α) mRNA was observed in the groups treated with DOX at 3 mg/kg/week. Together, these genomic cardiomyopathy indicators were significantly altered between 2 and 4 weeks of treatment and remained altered after 6 weeks of treatment. Furthermore, they were not affected by EPS and DZR alone (data not shown). It is important to note that only two out of six animals survived 6 weeks dosing of DOX 3 mg/kg/week.

### Perturbation of Rat Heart microRNAs during Chronic DOX Treatment

Expression profiles of 518 rodent microRNAs were generated by using a low density array qPCR platform (TLDAs). Approximately 370 microRNAs gave a measurable signal below the arbitrary cut-off set at 35 qPCR cycle times (Ct), with no major distinction between controls and treated samples. The administration of 4 weekly doses of DOX 3 mg/kg/week led to increased levels of 17 microRNAs and decrease levels of 8 microRNAs (p value below 0.05) (Table 1). Notably, a subset of those was consistently modulated at the earlier 2 weeks time point (fold change >1.5 are italicized in Table 1).

**Table 1:** Chronic DOX treatment (3 mg/kg/week) alters levels of 25 microRNAs from week 2 onwards.

	2 weeks		4 weeks	
DOX 3 mg/kg/week	FC	P value	FC	P value
*mmu-miR-367^#^*	4.58	1.97E−01	74.69	**1.76E**−**06**
mmu-miR-215*^#^*	1.11	9.41E−01	51.42	**7.45E**−**03**
*mmu-miR-216b^#^*	42.58	**4.54E**−**03**	24.17	**2.92E**−**03**
*mmu-miR-383^#^*	2.22	5.69E−01	21.5	**5.97E**−**04**
*mmu-miR-692^#^*	1.79	5.16E−01	17.71	**9.47E**−**03**
*mmu-miR-135b*	1.74	1.07E−01	12.69	**4.04E**−**02**
mmu-miR-667	1.49	3.08E−01	7.19	**1.45E**−**02**
mmu-miR-298	−1.44	1.83E−01	3.67	**1.69E**−**02**
mmu-miR-145*	1.6	8.87E−02	3.42	**1.09E**−**02**
mmu-miR-208b*^#^*	1.09	7.94E−01	3.23	**3.05E−03**
mmu-miR-21*	1.16	7.36E−01	3.21	**4.26E**−**02**
*mmu-miR-877*	1.55	1.79E−01	2.74	**2.33E**−**02**
mmu-miR-709*^#^*	1.04	8.38E−01	2.51	**7.28E−03**
mmu-miR-708*^#^*	1.11	7.08E−01	2.34	**1.57E**−**02**
mmu-miR-31	−1.02	9.68E−01	2.32	**3.95E**−**02**
mmu-miR-34c*^#^*	1.02	9.55E−01	2.25	**3.06E**−**02**
rno-miR-29b-2*	−1.1	6.84E−01	2.01	**3.41E**−**02**
mmu-miR-218-2*^#^	1.03	9.79E−01	−8.61	**3.45E−03**
*mmu-miR-434-5p*	−3.21	3.29E−01	−6.14	**4.81E**−**02**
rno-let-7e*	1.49	6.00E−01	−3.95	**1.84E**−**02**
*mmu-miR-337-5p*	−2.86	2.30E−01	−3.76	**4.37E**−**02**
mmu-miR-335-3p	1.43	1.33E−01	−2.52	**3.47E**−**02**
mmu-miR-384-3p	1.07	7.52E−01	−2.38	**4.32E**−**02**
mmu-miR-221	1.37	1.15E−01	−2.08	**1.85E**−**02**
rno-miR-1*	−1.16	8.05E−01	−2.01	**3.29E**−**02**

Variation of cardiac microRNA levels versus vehicle are reported for animals treated with DOX 3 mg/kg/week for 2 and 4 weeks. Values were calculated via the relative quantification (ΔΔCt) method by using the mammalian U6 snRNA as a normalizer. MicroRNAs showing same trend at 2 and 4 weeks are italicized. # indicates microRNAs selected for further analysis. Significant P values (<0.05) are in bold. FC  =  fold change.

### MiR-367, miR-215, miR-216b, miR-208b and miR-34c are Specifically Dysregulated by Chronic DOX Treatment

**Figure 3:**
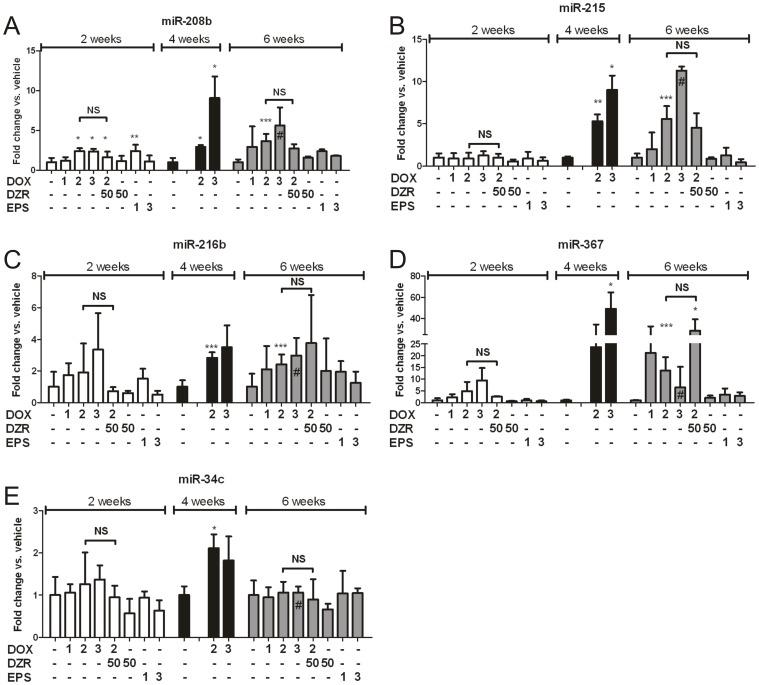
Relative quantification of DOX-responder microRNAs in rat heart across all groups. Relative quantification of (A) miR-208b, (B) miR-215, (C) miR-216b, (D) miR-367 and (E) miR-34c in DOX, DOX + DZR, EPS groups, normalized versus vehicle treated animals. Expression levels were measured by single assay qPCR (n = 3, except #, n = 2). DOX: Doxorubicin, DZR: dexrazoxane, EPS: etoposide; numbers indicate the weekly dose of each compound in mg/kg/week. Empty spaces represent non-sampled animals. The vehicle treated is the first column of each time-point. The animals used in this experiment were distinct from the ones represented in Table 1. Error bars represent SD. T-test results are indicated by asterisks for significant DOX-treated groups vs. their own vehicle-treated, unless otherwise specified by horizontal range bars; *P<0.05, **P<0.01, ***P<0.005, NS = Non-Significant).

A subset of ten microRNAs whose expression was altered by chronic DOX treatment was selected based on their lowest p values (Table 1) and evaluated their expression profile in additional individual animals from the same DOX treatment groups (originating from the same *in vivo* study) using single Taqman assays (Figure S2). Five of these microRNAs (miR-208b, miR-215, miR-216b, miR-34c and miR-367) displayed a consistent dose-dependent response to DOX at 2 and 4 weeks and were thus chosen as candidates for further expression profiling across the larger *in vivo* study treatment groups including co-treatment of DOX with DZR and treatment with EPS only (Figure 3).

Following two weeks treatment with DOX alone, a slight up-regulation of miR-208b, mir-216b and miR-367 was observed in the heart of most animals. Following 4 weeks of treatment with DOX alone, the level of all five microRNA candidates were significantly increased and dose dependency was observed for miR-208b, miR-215, miR-216b and miR-367. Following 6 weeks of treatment with DOX alone, the levels of miR-208b, miR-215, miR-216b and miR-367 were significantly increased. Dose dependency was difficult to assess at the 6 week time point since only 2 animals survived in the high dose group.

Following treatment with DZR alone for 2 and 6 weeks, none of the five candidate microRNAs was affected. After treatment with the combination of DOX at 2 mg/kg/week and DZR at 50 mg/kg/week, the level of miR-208b, miR-216b and miR-367 was either slightly increased or not affected. For all five candidate microRNAs, the level was lower when compared to the group of animals treated for 2 weeks with DOX alone at 2 mg/kg/week. Following 6 weeks of treatment with the same DOX/DZR combination, the level of miR-208b, miR-216b and miR-367 was increased. However, this level was lower for miR-208b and miR-216b when compared to the group of animals treated 6 weeks with DOX alone at 2 mg/kg/week and higher for miR-367.

Following 2 and 6 weeks of treatment with EPS, the expression level of the 5 microRNA candidates was minimally affected and considered as non statistically significant since no dose and time dependency was observed (although these changes might still be biologically relevant). Together, these data suggested that the variation of these five microRNAs was DOX-specific.

In summary, chronic treatment with DOX at 3 mg/kg/week for 2 weeks led to increases in miR-208b, miR-216b and miR-367, with fold change intensities comparable to those shown by genomic cardiomyopathy indicators (Ankrd/Carp, Nppb, Myh7 and Myh6) (Figure 2 and Table 1), suggesting that microRNAs may enhance the predictivity of established genomic indicators of drug-induced cardiomyopathy (e.g. myosin genes, troponins, natriuretic peptides) when assessing the potential for drug-induced cardiotoxicity.

### DOX Induced Up-regulation of miR-216b and miR-367 Earlier than Tissue Lesions as Observed by Histopathology

**Figure 4:**
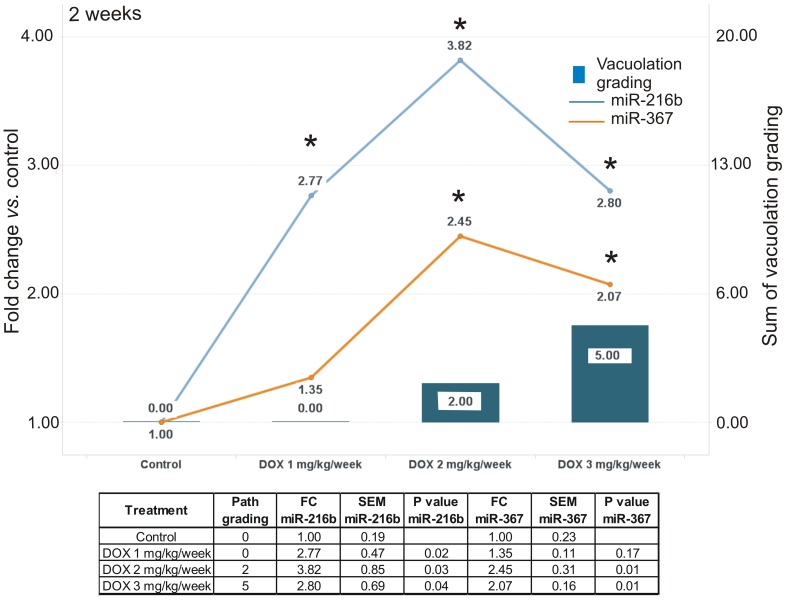
DOX-induced upregulation of miR-216 and miR-367 expression precedes the detection of overt histopathological lesions in cardiac tissue. Blue bars show cumulative vacuolation grade. X axis shows the DOX regimen in mg/kg/week received by the animals at 2 weeks. Y axes report cumulative histopathological scores and microRNA fold change vs. untreated cardiac tissues (normalized at value 1). Path grading  =  cumulative vacuolation score. FC  =  fold change. SEM  =  standard error on the mean.

We then compared the variation of DOX-specific microRNA perturbations with the severity of cardiac tissue lesions induced by drug (cytoplasm vacuolation) in order to evaluate their sensitivity. Increasing doses of DOX led to higher cumulative vacuolation grading with time (Results S1 and Table S2), as well as progressively larger fold changes for microRNAs miR-208b, miR-216b and miR-367 versus vehicle. Notably, treatment with DOX 1 mg/kg/week during 2 weeks did not cause any detectable vacuolation, but showed a statistically significant increase of miR-216b (Figure 4). Treatment with DOX at 2 and 3 mg/kg/week for 2 weeks showed a statistically increase of miR-216b and miR-367 (Figure 4). Interestingly, we observed that miR-208b profile across treatment groups had the best statistically significant correlation score in comparison to the severity of vacuolation (Spearman R squared  = 0.76, p = 4.45E-04) (Results S1 and Table S3). Therefore miR-208b may be implicated in the pathology mechanism of the vacuolation. These data suggest that DOX-induced perturbations of cardiac microRNA expression can precede overt tissue histopathology and that specific microRNAs may be useful as genomic indicators of drug-induced cardiac toxicity.

### MiR-34c Regulates Sipa1 mRNA at the Post-transcriptional Level and is Involved in Autophagy Process

**Figure 5:**
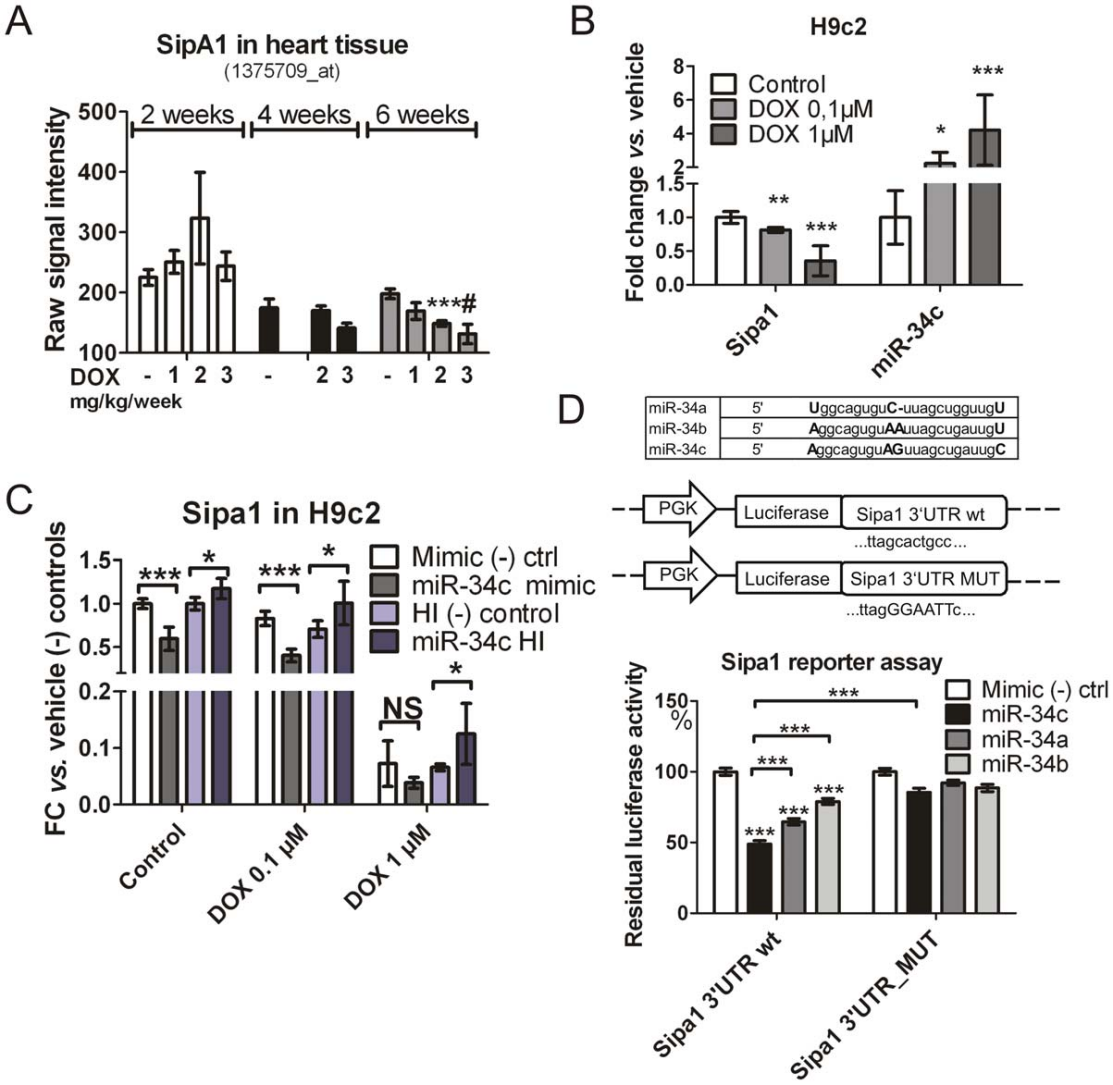
miR-34c directly controls DOX-induced Sipa1 mRNA decrease. (A) Sipa1 mRNA raw expression values decreased in the heart of rats treated with DOX. #, n = 2. (B) DOX treatment for 24 h caused a decrease of Spa1 mRNA and an increase of miR-34c in cardiac myoblast cells (H9c2). (C) H9c2 endogenous Sipa1 mRNA was decreased by transfection of miR-34c mimic, and increased using a miR-34c hairpin inhibitor (HI). Transfection with miR-34c mimic and inhibitor respectively exacerbated and rescued Sipa1 mRNA levels in H9c2 treated with DOX 0.1 and 1 µM overnight in comparison to negative controls. (D) Alignment of mammalian miR-34 family. Capital letters indicate mismatch in the sequence. Sipa1 3′-UTR wt and MUT construct: 12 nt surrounding the predicted seed are shown. HEK 293 cells were co-transfected with pmiR-GLO-Sipa1 and the indicated miRNA mimic or a *C. elegans* negative control mimic. Averaged and normalized Renilla luciferase signal was obtained from 3 independent experiments, each run in quadruplicate. Y axis represents percentual residual luciferase activity in the indicated conditions. Mutant 3′ UTR restores luciferase activity in Sipa1/miR-34c. *P<0.05, **P<0.01, ***P<0.005.

Out of the 7804 mRNA targets for DOX-inducible microRNAs predicted by Microcosm, 132 (1.7%) mRNAs showed the expected profile of anti-correlated expression relative to its putative targeting microRNA (>1.5 fold change at DOX 3 mg/kg/week for 4 weeks *vs.* vehicle) (Table S4). Since this low prediction rate could have been obtained by statistical chance, the putative microRNA-mRNA interaction of miR-34c/Sipa1was selected for further experimental evaluation. Treatment with DOX at 1, 2 and 3 mg/kg/week showed a dose-dependent decrease of signal-induced proliferation-associated 1 (Sipa1) and a dose-dependent increase of miR-34c (Figure 5A). Similarly, the treatment of H9C2 rat myoblasts with DOX at 0.1 and 1 µM for 24 h showed a decrease of Sipa1 mRNA and an increase of endogenous miR-34c (Figure 5B). The transfection of miR-34c mimics in H9c2 showed a statistically significant decrease of Sipa1 mRNA, as the transfection of miR-34c hairpin inhibitor (HI) showed a statistically significant increase of Sipa1 mRNA (Figure 5C). Notably miR-34c over-expression exacerbated Sipa1 down-regulation induced by DOX, while the transfection with mir-34c hairpin inhibitor significantly rescued the expression (Figure 5C), suggesting that miR-34c is mediating the DOX-induced regulation of Sipa1. Finally, to confirm whether the regulatory effect of miR-34c on the levels of Sipa1 mRNA was direct, 2 luciferase-based reporter expression vectors (pmiR-GLO-Sipa1 WT and mutated) were generated by cloning a 200 nucleotide region surrounding the predicted microRNA seed from each of their 3′-UTR regions downstream of a firefly luciferase reporter gene. The co-transfection of the Sipa1 reporter with miR-34c in HEK 293 cells showed a significant decrease in the luciferase signal. This decrease was observed to a lower extent with other members of the miR-34 family (Figure 5C) suggesting that miR-34c interacted directly and specifically with the 3′-UTR of Sipa1. Therefore miR-34c is directly implicated in the DOX-induced modulation of Sipa1 mRNA regulation.

**Figure 6:**
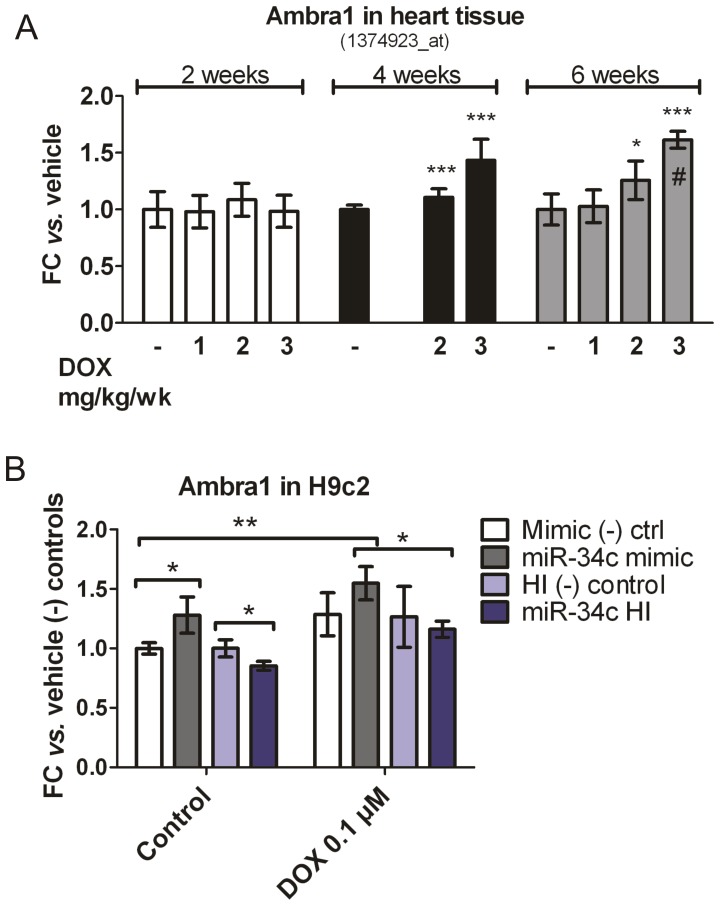
Ambra1 expression was induced by DOX treatment *in vivo* and miR-34c could control its endogenous expression levels in H9c2 cardiac myoblasts. (A) Fold change of Ambra1 probe set in rat heart tissue treated with DOX. Fold change and statistical significance were assessed *vs.* each vehicle group. n = 4 to 6 (except #, n = 2) (B) Endogenous levels of Ambra1 were measured after miR-34c over-expression (miR-34c mimic) or inhibition (miR-34c HI) in absence of presence of DOX 0.1. Fold change value were normalized *vs.* the respective negative transfection controls in the untreated condition (n = 3). *P<0.05, **P<0.01, ***P<0.005. FC  =  fold change.

We have also used the rat myoblast H9c2 cell line to assess the DOX-induced autophagy process through the regulation of Ambra1 mRNA (Figure 6). Transfection of miR-34c mimics showed a statistically significant increase of Ambra1 mRNA. Transfection of miR-34c hairpin inhibitor showed a statistically significant decrease of Ambra1 mRNA. Treatment with DOX at 0.1 µM for 16 h in the presence of miR-34c mimics showed a statistically significant increase of Ambra1 mRNA. Treatment with DOX at 0.1 µM for 16 h in presence of miR-34c hairpin inhibitor showed a statistically significant decrease of Ambra1 mRNA when compared to the presence of miR-34c mimics. The extent of Ambra1 mRNA regulation is slight but comparable between the *in vivo* systems and the H9c2 (Figure 6A and B). Treatment with DOX in H9c2 did not affect the level of miR-208b ruling out a potential implication of miR-208b with autophagy in this cellular model.

## Discussion

In the present study 25 microRNAs were found to be dysregulated by pharmacological intervention with DOX in the rat heart. A subset of microRNA candidates were further investigated and associated to their putative targets.

The chronic weekly intravenous administration of DOX was designed to trigger cardiomyopathy symptoms within a 6 week time-frame allowing us to identify mRNAs and microRNAs that are altered in the early stages of the drug-induced cardiac remodeling. Cardiac toxicity at a cumulative dose of 12 mg/kg (3 mg/kg/week for 4 weeks) was confirmed by histopathological lesions and altered expression of genomic indicators associated to contraction performance (Myh6, Myh7) and heart failure (Ankrd1/CARP, Nppb) (Figure 2) [28], [29], [30], [31], [32]. In most of the cases the dysregulation of these genes reached a significantly higher level between 2 and 4 weeks of the DOX 3 mg/kg/week dosing regimen. Additionally, 2 weeks treatment with DOX at 2 mg/kg/week onward caused cytoplasm vacuolation to occur with increasing severity.

We profiled hundreds of microRNAs via TLDAs in the heart of rats that showed clear signs of toxicity (DOX 3 mg/kg for 4 weeks), and found that 25 microRNAs were dysregulated with p values below 0.05. Seven out of 25 microRNAs (miR-367, miR-216b, miR-383, miR-692, miR-135b, miR-145*, miR-877, miR-434-5p and miR-337-5p), were already modulated after 2 weeks of DOX at 3 mg/kg/week (Table 1). Comparable changes for many of these microRNAs were confirmed in additional animals, and a subset of five microRNAs (miR-208b, miR-215, miR-216b, miR-367 and miR-34c) and were largely unaffected by the direct Top2 inhibitor etoposide (EPS) (Figure 3). However DOX-induced up regulation of these microRNAs was not lessened by co-treatment with DZR, suggesting that these were not involved in the molecular pathways triggered by DZR.

It is noteworthy that miR-21 and miR-146a, previously linked to DOX-induced apoptosis in cardiomyocytes *in vitro* [33], [34] or by single administration of a high dose of DOX *in vivo* [33], were not among the microRNAs regulated by our chronic DOX dosing regime in Sprague Dawley rats. Similarly, miR-1 and miR-133, known to have alternative effects on oxidative stress-induced apoptosis in myocytes [35], the former being pro-apoptotic and the latter anti-apoptotic, were not altered in the present study. These discrepancies may derive from several key differences in the model systems and study designs used. Importantly, primary cells or cell lines treated *in vitro* are not comparable to the whole cardiac tissue when exposed to metabolized systemic DOX and the work of Horie *et al.* explores the acute effect of a single high dose of DOX, which contrasts to the chronic regimen administered here. Our present study describes global changes in microRNAs during chronic drug-induced cardiac toxicity, in a way that closely resembles the safety signals observed in a clinical setting.

In our study miR-215, part of the miR-192/miR-215 cluster, and miR-34c, involved in DNA damage mediated proliferation arrest [36], [37], [38], [39], were up-regulated, consistent with the pharmacological effect of DOX. However miR-215 and miR-34c were not significantly up-regulated by EPS administration, suggesting that this compound may not trigger a p53 response under these experimental conditions.

Interestingly miR-215, up regulated by DOX in this study, was also linked to p53 mediated cell cycle arrest [37], [38], consistent with the pharmacology of DOX and partially replicating expression changes of miR-34c upon treatment, thus strengthening the biological relevance of our findings and consistency with previously published mechanisms [36].

MicroRNA-208b, encoded from the intron 28 of rat Myh7, is associated to maintenance of myocardial performance together with 2 other myomirs, i.e. miR-208a/miR-499, which play a pivotal role in the myosin balance [40]. Consistent with previous work, the DOX-induced expression of miR-208b parallels the modulation of expression of its host gene Myh7 (Figure S1B) and occurs in parallel to the appearance of vacuolation (Table S3). The concomitant decrease of miR-208a and Myh6 levels upon DOX treatment (Figure S1A) further support the importance of the regulatory role of myomiRs-myosin interactions in the myosin switch, which is associated with progressive structural changes and pathological cardiac remodeling [40].

Of particular interest is the predictive potential of microRNAs as early tissue indicators of drug-induced cardiac lesions. The lowest dose of DOX tested in our study (1 mg/kg/week for 2 weeks) did not cause detectable tissue vacuolation (Table S2). However, miR-216b was already up-regulated (>1.5 fold) under these conditions, and miR-367 concomitantly increased at the earliest time point (2 weeks) in the different treatment groups. Treatment with DOX at all doses and time points was associated with sustained up-regulation of these microRNAs, together with miR-208b and miR-215 at later time points. Further studies will be needed to investigate the potential of circulating microRNAs as indicators of DOX- induced cardiac injury.

In order to gain further insight into the cellular processes that may be modulated upon DOX-induced cardiac toxicity, high confidence target mRNAs of DOX-dysregulated microRNAs were identified by using the Microcosm prediction tool (Table S4). In particular, only the genes that had an anti-correlated change of expression at the mRNA level *vs.* the cognate microRNA were retained. The screening for putative targets of microRNAs in DOX treated animals, revealed that a number of DOX-responsive microRNAs may regulate mRNAs involved in cardiac tissue remodeling (data not shown), but we also speculated that the Sipa1 mRNA transcripts could be targeted by miR-34c directly. Sipa1 gene product is an activator of Rap GTPase and can influence proliferation rate and metastatic potential in various types of malignancies [41], [42], [43]. Moreover cAMP-mediated Rap signaling, and its modulators, played a role in significant aspects of cardiovascular physiology, including cardiomyocytes contraction and hypertrophy [44]. Importantly, Sipa1 mRNA showed an anti-correlated pattern with miR-34c in H9c2 cells treated with DOX 1 µM during 24 h (Figure 5B). Reporter assay experiments further support that Sipa1 mRNA is a target of the miR-34 family, in particular miR-34c (Figure 5). In addition, our observations in H9c2 myoblast cell line suggest that miR-34c could be involved in the stimulation of the positive autophagy regulator Ambra1, also in presence of DOX. In fact, miR-34c over expression showed a statistically significant increase of the endogenous levels of Ambra1 mRNA (both with and without DOX). Vice versa transfection of a miR-34c-directed hairpin inhibitor showed a statistically significant decrease of Ambra1 mRNA level. However, the functional relevance of this finding in autophagy regulation and the exact mechanism of this regulation will have to be elucidated. Further studies, such as gain- or loss-of-function *in vivo* investigations, will be required to elucidate the functional significance of altered Sipa1 expression for cardiac responses to DOX. Immunochemistry staining of autophagy markers like Ambra1 in heart tissues will also be required to elucidate the functional involvement of miR-215, miR-216b, miR-367, miR-208b and miR-34c in the DOX-induced autophagy process since they seem to be specifically associated to vacuoles appearance.

In conclusion, 25 microRNAs implicated in DOX-induced cardiac toxicity *in vivo* were identified. Among them, miR-216b, which was significantly regulated before overt toxicity, has the potential of a genomic indicator of cardiac toxicity.

Our study also provides the basis for understanding the global role of microRNAs in DOX-induced cardiac remodeling and toxicity, and provides evidence the direct miR-34c/Sipa1 functional interaction for the first time.

## Materials and Methods

### Rat *in vivo* Study and Histopathology Evaluation

This in vivo study was conducted in compliance with the Animal Welfare Act, and the Office of Laboratory Animal Welfare, after review and approval by the Covance site (Vienna, Virginia) Institutional Animal Care and Use Committee. Doxorubicin (Adriamycin, Lot No. 86G23FY, CAS# 23214-92-8), a generous gift from Adria Laboratories Inc. (Columbus, OH, USA), etoposide (ETOPOPHOS® Lot No. 5E04155, CAS# 117091-64-2) was purchased from Bristol-Myers Squibb (Princeton, NJ, USA) and dexrazoxane (Zinecard® Lot No. ADR074B, CAS# 24584-09-6) purchased from Pharmacia/Pfizer (Kalamazoo, MI, USA) were administered once weekly as follows: 11 week old male Crl:CD(SD) rats received DOX or EPS via intravenous injection once a week for 2, 4, or 6 weeks based on Table S1 at a dose volume of 2 mL/kg. Animals in the DZR groups were given a 10 mL/kg intraperitoneal injection of DZR followed (30±2 minutes later) by a 2 mL/kg IV injection of 0.9% sodium chloride or DOX. The intravenous (iv) route of administration for DOX and EPS and the intraperitoneal (ip) route for DZR respectively, were selected to match the clinical route of administration in humans. More details are provided in the Material and Methods S1 and in a forthcoming publication from Gerrish et al. The experimental strategy is summarized in Figure 1A.

### Heart Tissue RNA Extraction

Total RNA was obtained from 50–100 mg frozen cardiac tissue homogenized in Trizol, according to manufacturer instructions (Invitrogen, Life Technologies, Carlsbad, CA). Long and short RNA fractions were separated via affinity resin column during clean up, according to the manufacturer instructions (miRNeasy Mini Kit, Qiagen, MD). Short RNA was quantified by absorbance at 260 nm using a Nanodrop (Thermo Scientific, Wilmington, DE), and the quality and integrity were determined using Small RNA chips (Agilent Technologies, Santa Clara, Ca) and stored at −80°C until analysis.

### Gene Expression Profiling and Analysis

GeneChip experiment was conducted in the BMD Genechip laboratories (Novartis, BS) on Rat Genome Rat230 2.0 Array (Affymetrix, Inc.). Target preparation was performed with a starting amount of approximately 1 µg of total RNA unless otherwise specified using the Affymetrix GeneChip HT One-Cycle Target Labelling and control reagent according to manufacturer’s instruction (Affymetrix, Inc.). cRNA size distribution (before and after fragmentation) was confirmed by agarose-gel electrophoresis. An amount of biotinylated cRNA of approximately 10 µg for each sample was hybridized for approximately 16 hours at 45°C on an array. The array was washed and stained on Affymetrix Fluidics Workstation 450 and scanned on Affymetrix Scanner 3000 according to manufacturer’s technical manual. The scanned image was converted into numerical values of the signal intensity (Signal) and into categorical expression level measurement (Absolute Call) using the Affymetrix MAS 5.0 software. The software scaled the average intensity of each chip to a target intensity of 150. The microarray data are available in ArrayExpress under accession number E-MTAB-1168.

### Amplification of microRNAs via Quantitative PCR

Using the Taqman Low Density Array (TLDA) technology (Applied Biosystems, Life Technologies, Carlsbad, CA), about 500 rodent microRNAs were profiled without any pre-amplification step, using 700 ng of small RNA according to the manufacturer instructions (Applied Biosystems, Life Technologies, Carlsbad, CA). Sufficient RNA amount for the TLDA cards was obtained for 3 out of 6 animals receiving DOX 3 mg/kg/week for 4 weeks, and for 6 out of 6 receiving DOX 3 mg/kg/week for 2 weeks. An arbitrary cut-off at 35 Ct was set in order to exclude microRNAs out of the linear range of detection. Mammalian snRNA U6 was used as housekeeping gene for its expression was not affected by DOX treatment. In order to confirm the results of the TLDA, a subset of microRNAs were quantified using single Taqman assays in the 3 remaining animals treated with DOX 3 mg/kg/week for 4 weeks. All procedures were carried out according to manufacturer instructions (Applied Biosystems, Life Technologies, Carlsbad, CA) using a 7900HT Fast Real-Time PCR System.

### Data Analysis

MicroRNA data obtained from the TLDAs were quality checked and analyzed using Statminer plugin (Integromics, Madrid, Spain) for TIBCO Spotfire (TIBCO Software, Palo Alto, CA). Average fold changes and standard deviations were calculated and plotted using GraphPad Prism 5.00 (GraphPad Software, San Diego California USA). Lists of putative targets for each DOX-regulated microRNA were obtained from Microcosm [45]. Raw intensity values for each probe set were generated using RMA algorithm, using Genespring GX 7.3 (Agilent Technologies, Santa Clara, CA). Gene lists were filtered for raw expression (>80 raw signal in at least 50% of arrays) and anti-correlation with the microRNA (absolute fold change >1.3 or 1.5 in animals receiving DOX 3 mg/kg for 4 weeks *versus* control treated). Candidates passing this cut-off were used to build networks of up- or down-regulated genes with their putative cognate microRNAs. P values were generated via unpaired, two-tailed Student t-test and were considered significant below 0.05. Phenotypic anchoring and Spearman correlation analysis were realized using TIBCO Spotfire (TIBCO).

### Cell Culture and DOX Treatment

HEK 293 (CRL-1573, ATCC) and rat myoblastic cells H9c2 (CRL-1446, ATCC) were cultured in MEM and DMEM respectively, supplemented with 10%v/v heat-inactivated fetal bovine serum (Invitrogen). DOX was purchased from Sigma, diluted in DMSO to a stock concentration of 10 mM and stored at −20°C. DOX treatment of H9c2 cells (n = 3) was independently repeated 3 times (total of 9 replicates). DOX was added cells transfected with microRNA reagents after 4 hours, buy replacing transfection medium with fresh growth medium supplemented with DOX at the indicated concentrations.

### MicroRNA Target Luciferase Reporter Assay

Two-hundred nucleotides surrounding the microRNA predicted seed site in the 3′-UTRs of either murine Tnni3k or Sipa1 were synthesized (GeneArt, Invitrogen, Carlsbad, CA) and cloned into a pmiR-GLO vector (Promega, Madison, WI) downstream of the luciferase gene via a XbaI-SacI restriction site to generate the pmiR-GLO-Sipa1 wt expression vector. The pmiR-GLO expression vector contains both the luciferase and the Renilla genes under the control of different promoters. Complete insert sequences are listed in Material and Methods S1 section. Stratagene “Quick change” kit was used to introduce a 6 nt mutation in the miR-34c predicted seed site to generate the pmiR-GLO-Sipa1 mutant and the pmiR-GLO-Tnni3k mutant expression vectors.

Sipa1 mutagenesis Primer1∶5′- CTG CGC TGA GGC GCG TCT TAG **GGA ATT** CCC CTC TTC CCA GCC CAT TTG -3′. Sipa1 mutagenesis Primer 2∶5′- CAA ATG GGC TGG GAA GAG GGG **AAT TCC** CTA AGA CGC GCC TCA GCG CAG -3′.

### Transfection of HEK 293

HEK 293 cells were plated in 24 wells plates 24 hours prior to co-transfection in antibiotic-free medium. Cells (2×10^5^ per well) were co-transfected with the reporter constructs (20 ng/well) and synthetic mmu-miR-34a, b or c (40 or 80 nM) mimics (Dharmacon, Lafayette, CO) in serum-free medium, using Lipofectamine 2000 (Invitrogen, Carlsbad, CA). A *C. elegans* microRNA mimic with no affinity for mammalian targets was used as negative control. Dual-GLO Luciferase Assay System (Promega, Madison, WI) was used to assess luciferase relative luminescence, according to manufacturer instructions.

## Supporting Information

Figure S1 **MicroRNA-208a and 208b are regulated similarly to their hosting transcripts (Myh6 and Myh7 respectively) upon DOX treatment (n = 3).** (A) Myh6 and miR-208a (n = 3) and (B) Myh7 and miR-208b fold changes vs. control in DOX 3 mg/kg/week treated animals. (n = 3). *P<0.05, **P<0.01, ***P<0.005
(TIF)

Figure S2 **Single assay validation of TLDA data.** Ten microRNAs among the DOX 3 mg/kg/week 4 weeks dysregulated microRNAs (Table 1) were assayed in the remaining 3 animal tissues at the same dose and in 3 animals for each indicated dose and timepoint (2 and 3 mg/kg for 2 and 4 weeks). All except miR-709 and miR-692 confirmed the trends observed with the LDA-qPCR technique. Fold changes for the given doses are indicated vs. untreated animals. (A) miR-208b, (B) miR-215, (C) miR-216b, (D) miR-218-2*, (E) miR-34c, (F) miR-367, (G) miR-383, (H) miR-692, (I) miR-708 and (J) miR-709.
(TIF)

Table S1 **Study design.**
(XLS)

Table S2 **Atrial and ventricular vacuolation incidence increases with doses of DOX.** Microscopic histopathological findings. Severity of lesion is graded by numbers. Lo = 1 mg/kg/week regimen; Mid = 2 mg/kg/week regimen; Hi = 3 mg/kg/week regimen.
(XLS)

Table S3 **Spearman correlations of microRNAs levels and cumulative vacuolation severity in the heart tissue of rats treated with DOX.**
(XLS)

Table S4 **Number of Microcosm predicted targets of 16 out of 25 microRNAs, responding to DOX 3 mg/kg/week after 4 weeks.** Raw gene expression was filtered to be higher than 80 in at least 50% of the conditions at the cumulative dose of 12 mg/kg. The absolute fold change anti-correlated to the cognate microRNA was used to further reduce the lists at 2 different stringencies. Number of genes passing these filters is indicated for each signature-microRNA for which prediction was available.
(XLS)

Results S1 **Supporting results.**
(DOC)

Material and Methods S1 **Supporting Material and Methods.**
(DOC)
