# Supplementary figures and images for: Perturbation of microRNAs in Rat Heart during Chronic Doxorubicin Treatment
Source: PLoS One. 2012 Jul 31;7(7):e40395. doi: 10.1371/journal.pone.0040395 (PMC3409211; doi:10.1371/journal.pone.0040395)

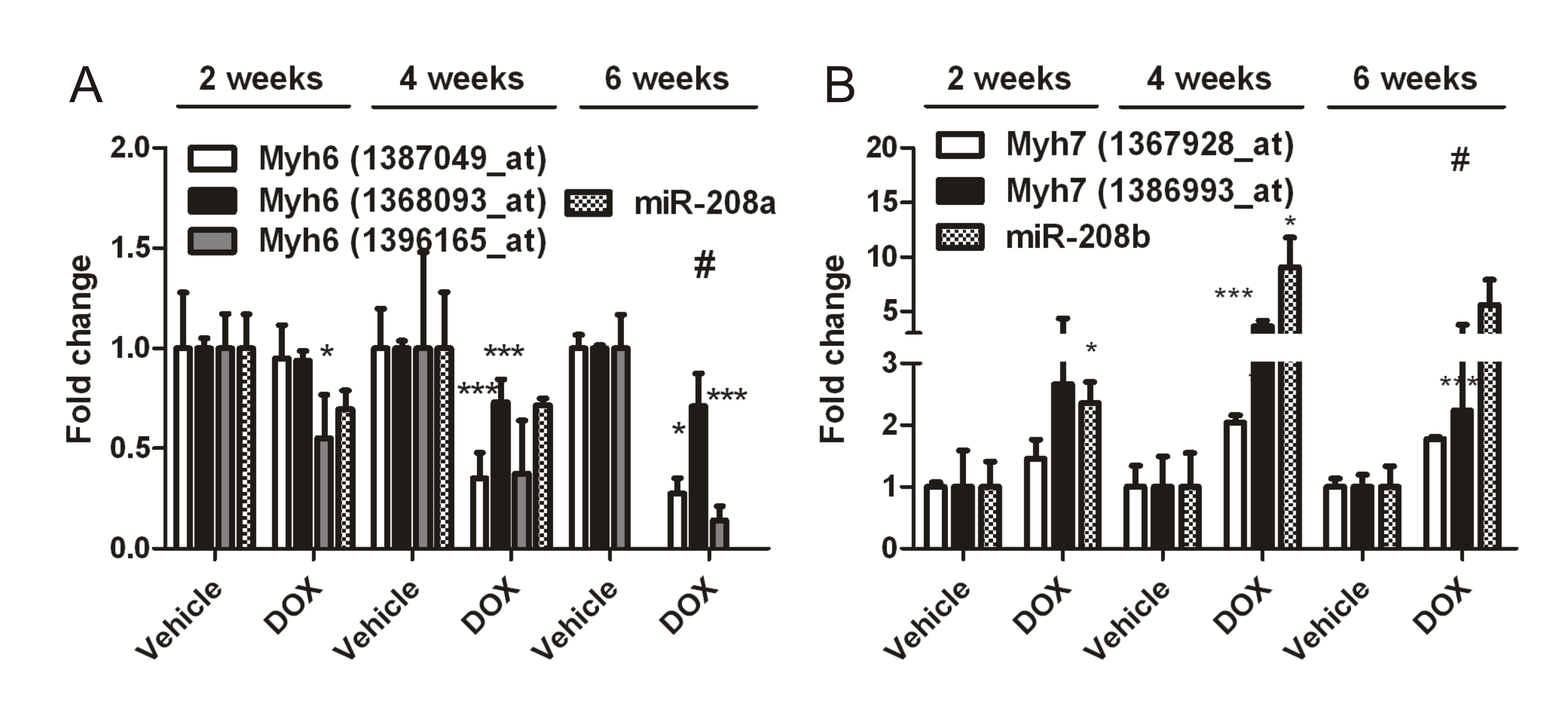

Supplement: Figure S1 — MicroRNA-208a and 208b are regulated similarly to their hosting transcripts (Myh6 and Myh7 respectively) upon DOX treatment (n = 3). (A) Myh6 and miR-208a (n = 3) and (B) Myh7 and miR-208b fold changes vs. control in DOX 3 mg/kg/week treated animals. (n = 3). *P<0.05, **P<0.01, ***P<0.005 (TIF) [file pone.0040395.s001.tif]

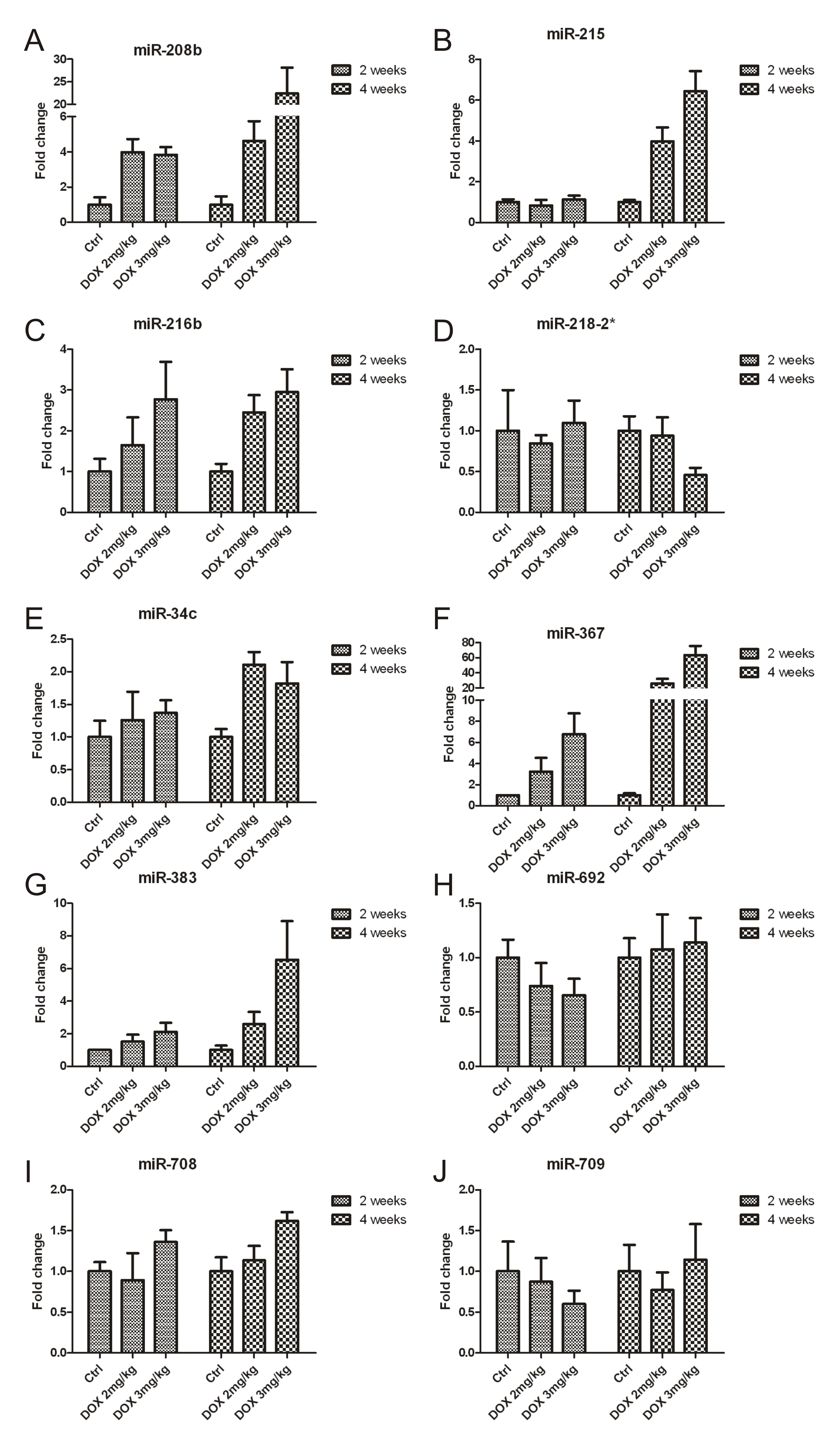

Supplement: Figure S2 — Single assay validation of TLDA data. Ten microRNAs among the DOX 3 mg/kg/week 4 weeks dysregulated microRNAs (Table 1) were assayed in the remaining 3 animal tissues at the same dose and in 3 animals for each indicated dose and timepoint (2 and 3 mg/kg for 2 and 4 weeks). All except miR-709 and miR-692 confirmed the trends observed with the LDA-qPCR technique. Fold changes for the given doses are indicated vs. untreated animals. (A) miR-208b, (B) miR-215, (C) miR-216b, (D) miR-218-2*, (E) miR-34c, (F) miR-367, (G) miR-383, (H) miR-692, (I) miR-708 and (J) miR-709. (TIF) [file pone.0040395.s002.tif]
