# Supplementary material for: Perturbation of microRNAs in Rat Heart during Chronic Doxorubicin Treatment
Source: PLoS One. 2012 Jul 31;7(7):e40395. doi: 10.1371/journal.pone.0040395 (PMC3409211; doi:10.1371/journal.pone.0040395)
Supplement: Results S1 — Supporting results. (DOC) [file pone.0040395.s007.doc]

# Results S1

**Body and organ weight**

Treatment with DOX caused dose-dependent decreases in mean body weight and weight gain in animals dosed with 2 or 3 mg/kg/week. Treatment with combination of DOX and DZR similarly induced a significant decrease in mean body weight gain. The changes in mean body weight and body weight gain appear to directly correlate with decreases in mean food consumption. A similar decrease was not noted in animals that received DZR or EPS alone, indicating that the decrease in mean body weight gain was attributable to DOX. Of the animals that survived to scheduled euthanasia and necropsy, the overall, significantly decreased heart weight parameters indicated a progressive, irreversible DOX-related toxic effect at dose levels of 2 or 3 mg/kg/week. Decrements in heart weight parameters were mitigated when DZR was given in conjunction with DOX illustrating protective properties of DZR (data not shown).

**Clinical chemistry**

Dose and time dependent alterations in hematology and serum chemistry were caused by DOX once weekly for 2, 4, or 6 weeks. There were minimal alterations in the clinical pathology from rats given DZR or EPS only (data not shown). In general, many of the alterations in rats given DOX in combination with DZR were of much smaller magnitude suggesting that it alleviates many of the side effects of DOX. More details will be given in a forthcoming publication by Gerrish *et al.*

**Histopathology scores of cytoplasm vacuolation in the heart**

DOX-induced cardiomyopathy is characterized by sarcoplasmic micro- and macro-vacuolation of the heart atrium and ventricle. These are generally categorized as minimal, mild, moderate and marked. In order to visualize the histopathological diagnoses we combined the pathology scores from the individual animals in each treatment group. There is a progressive increase in the cumulative pathology score in the hearts from DOX-treated animals (Table S 2).
