# Supplementary material for: Perturbation of microRNAs in Rat Heart during Chronic Doxorubicin Treatment
Source: PLoS One. 2012 Jul 31;7(7):e40395. doi: 10.1371/journal.pone.0040395 (PMC3409211; doi:10.1371/journal.pone.0040395)
Supplement: Material and Methods S1 — Supporting Material and Methods. (DOC) [file pone.0040395.s008.doc]

# Material and Methods S1

## Histopathology investigations of the heart

At necropsy, the heart was oriented with the left ventricle uppermost (i.e. aorta stem pointing towards operator) and then cut in half longitudinally. Each heart was longitudinally bisected from base to apex along the middle of the ventricular free walls to reveal right and left ventricles; right and left atria; both atrioventricular valves and the root of the aorta. One half of the bisected heart was placed in 4% formaldehyde/1% glutaraldehyde while the other half was flash-frozen for other studies. The fixed hemi-sections of heart were processed for methylmethacrylate plastic embedment, sectioned at 1-2 µm, stained with toluidine blue and examined histologically. Figure 1 shows representative histologic cardiac sections with evidence of cardiomyocellular injury, more specifically microvesicular vacuolation typical of doxorubicin cardiomyopathy [1,2] (Figure 1B and C). Vacuolar changes in ventricular and atrial cardiomyocytes were recorded separately and semi-quantitatively graded using a simple cardiomyopathy scoring method based on the frequency and severity of observed changes on a four step arbitrary scale from minimal to marked (1=Minimal, 2=Mild, 3=Moderate and 4=Marked).

## Murine Sipa1 3’-UTR region cloned for reporter assay

Wild type:

5’-cgttccaatctgaatctggacctgcttggaactgcctggcccctcagagcaactgggtcatactagtgcccttcctcaggacttcttccctgcgctgaggcgcgtcttagcactgccccctcttcccagcccatttggtggctaatgcctgtccctgtttgtaaatatcctgtaaagaaaaggagacatcagagtttaaaaaaaagaaacaacaagaagaagcaaacaactct -3’

MiR-34 seed mutant:

5’- cgttccaatctgaatctggacctgcttggaactgcctggcccctcagagcaactgggtcatactagtgcccttcctcaggacttcttccctgcgctgaggcgcgtcttagGGAATTcccctcttcccagcccatttggtggctaatgcctgtccctgtttgtaaatatcctgtaaagaaaaggagacatcagagtttaaaaaaaagaaacaacaagaagaagcaaacaactct

Reference List

1. Herman EH, el-Hage AN, Ferrans VJ, Ardalan B (1985) Comparison of the severity of the chronic cardiotoxicity produced by doxorubicin in normotensive and hypertensive rats. ToxicolApplPharmacol 78: 202-214.

2. Bertinchant JP, Polge A, Juan JM, Oliva-Lauraire MC, Giuliani I, et al. (2003) Evaluation of cardiac troponin I and T levels as markers of myocardial damage in doxorubicin-induced cardiomyopathy rats, and their relationship with echocardiographic and histological findings. Clin Chim Acta 329: 39-51.
